# Deep Learning Techniques for Automatic Detection of Embryonic Neurodevelopmental Disorders

**DOI:** 10.3390/diagnostics10010027

**Published:** 2020-01-07

**Authors:** Omneya Attallah, Maha A. Sharkas, Heba Gadelkarim

**Affiliations:** Department of Electronics and Communications, College of Engineering and Technology, Arab Academy for Science and Technology and Maritime Transport, Alexandria 1029, Egypt; msharkas@aast.edu (M.A.S.); heba.mehery@gmail.com (H.G.)

**Keywords:** deep learning, convolution neural networks (CNNs), machine learning, embryonic neurodevelopment disorders, MRI imaging

## Abstract

The increasing rates of neurodevelopmental disorders (NDs) are threatening pregnant women, parents, and clinicians caring for healthy infants and children. NDs can initially start through embryonic development due to several reasons. Up to three in 1000 pregnant women have embryos with brain defects; hence, the primitive detection of embryonic neurodevelopmental disorders (ENDs) is necessary. Related work done for embryonic ND classification is very limited and is based on conventional machine learning (ML) methods for feature extraction and classification processes. Feature extraction of these methods is handcrafted and has several drawbacks. Deep learning methods have the ability to deduce an optimum demonstration from the raw images without image enhancement, segmentation, and feature extraction processes, leading to an effective classification process. This article proposes a new framework based on deep learning methods for the detection of END. To the best of our knowledge, this is the first study that uses deep learning techniques for detecting END. The framework consists of four stages which are transfer learning, deep feature extraction, feature reduction, and classification. The framework depends on feature fusion. The results showed that the proposed framework was capable of identifying END from embryonic MRI images of various gestational ages. To verify the efficiency of the proposed framework, the results were compared with related work that used embryonic images. The performance of the proposed framework was competitive. This means that the proposed framework can be successively used for detecting END.

## 1. Introduction

Neurodevelopmental disorders (NDs) are major concerns threatening pregnant women, parents, and clinicians caring for healthy infants and children [1]. NDs are an assembly of deficiencies that affect the natural development of the central nervous system. They embrace defects that disturb the developmental function of the brain, which could lead to apparent neuropsychiatric complications, learning difficulties, language or non-verbal communication problems, or motor function disability [2]. The healthy growth of the central nervous system relies on complicated dynamic processes with several locative and temporal elements throughout pregnancy. NDs can initially start through embryonic development due to genetic or other reasons that affect embryonic parental life within or outside the uterus [3]. Up to three in 1000 pregnant women have embryos with brain defects (Griffiths et al., 2017) [4]. Therefore, the early detection of embryonic neurodevelopmental disorders (ENDs) is essential. It will allow clinicians to accurately diagnose the brain defect before the infant is born and decide the suitable treatment and observation plan. Parents will be well prepared with the type of disorder and how to deal with it. This will improve the quality of diagnosis and health management. It will also reduce chances of the progression of the neurological disorder after the birth of the embryo.

Magnetic resonance imaging (MRI) is a widely used medical instrument to non-invasively evaluate and observe the developmental condition of the embryonic brain in utero (Levine et al., 2003) [5]. Embryos MR images enable doctors to identify brain anomalies in a primary phase of development. Detecting brain defects from these images is very difficult and challenging. This is because embryonic movements and the tiny sizes of embryonic brains present a major problem during the imaging process. Moreover, for adults’ brain imaging, a particular radiofrequency (RF) skull coil is attached closely to the individual’s head. However, for embryonic MRI scanning, the coils are allowed to be inserted on the parental body and not near to the structure of attention (which is the embryonic brain), which render the image quality. Furthermore, the dissimilarities in tissue contrast seen in utero for older gestational age (GA) presents one more challenge [6]. Nevertheless, of these challenges existing in embryonic MRI imaging and detection of embryonic neurodevelopmental disorders (ENDs), there is significantly huge potential for ongoing research growth on the use of machine and deep learning approaches in this field to facilitate the detection and classification of ENDs.

Machine learning (ML) approaches are used extensively in medical applications due to their interesting capability for extracting valuable information from medical datasets [7]. Previous work done in embryonic neurodevelopmental disorder classification (ENDC) is very limited [8,9,10,11]. These articles applied standard machine learning methods to MRI images for feature extraction and classification of embryonic neurodevelopmental disorders. Feature description and extraction of these methods are handcrafted and depend on delineation of brain structures, which is arduous and prone to inter- and intra-rater inconsistency, or compound pre-processing of MRI images. It is also time-consuming and needs high computational cost [12,13]. Deep learning approaches, which are another family of machine learning methods, attracted many researchers working in the medical field in recent years [14]. They are preferred to standard machine learning approaches as they need small or no image processing procedure. Deep learning methods have the ability to deduce an optimum demonstration from the raw images without image enhancement, segmentation, and feature extraction processes, leading to a better classification process and lower complexity [15]. For these reasons, deep learning algorithms are more suitable for detecting and classifying ENDs.

In this paper, a new framework based on deep learning algorithms is proposed for detecting ENDs. This problem is very complex and not easily solved due to several reasons, starting from imaging of the embryo brain and processing of such types of images, and ending with segmenting the brain, detecting the abnormality, and classifying it. Segmenting embryonic brain from MRI images is significantly more difficult than segmenting adult MRI brain images. This is due to the high variation in size and anatomical shape during the embryo growth in the utero, the motion artefacts, and variation in the fetal orientation, while the cerebrospinal fluid (CSF) and the white matter (WM) generate a partial volume (PV) problem [16,17]. All of these challenges harden the detection and classification process of embryonic neurodevelopmental disorders. On the other hand, classifying neurodevelopmental disorders of the brain of the embryo was not extensively investigated, and very limited work was done to solve this problem. Such related work is limited to References [8,9,10,11] only. To our own knowledge, this is the first study that uses deep learning techniques to detect ENDs. Previous work that used deep learning methods focused on segmenting embryonic normal brains only but not detecting the embryonic brain disorders. The framework depends on transfer learning and deep feature fusion. This framework uses raw embryo brain images without the need for imaging processing techniques such as image enhancement, segmentation, and handcrafted feature extraction methods which were used in the previous related work with several limitations, leading to a better classification process and lower complexity [15]. The proposed framework firstly uses the raw images to construct three deep convolution neural networks (DCNNs) of different architectures to detect ENDs. Afterward, it extracts deep features from the three DCNNs to build three support vector machine classifiers individually trained with each deep feature of the three networks. Next, due to the high feature dimensional space of the deep features extracted from the three networks, principle component analysis (PCA) is employed to reduce their dimension individually. Finally, it investigates the effect of fusing multiple deep features to construct a detection model for ENDs and selects the best combination of deep features which influence the results. In order to test the performance of the proposed framework, the results are compared with related work made for ENDs using machine learning techniques.

## 2. Materials and Methods

### 2.1. Dataset

The embryonic brain dataset employed in this article is called the embryonic brain atlas [18]. It included 227 images of embryonic MRI images (113 were healthy and 114 had neurodevelopmental disorders) with GA ranging between 16 and 39 weeks. Masses of T_2_ weighted MRI images in coronal, axial, and sagittal planes were acquired with a single-shot, half-Fourier, Rapid Acquisition with Relaxation Enhancement (RARE) sequence technique. Because of the continuous movements throughout the observation, the acquisition process for every single image acted as a guide for the subsequent scanning procedure. An ordinary scanning process utilizes an echo time T_E_ of 60 ms, an echo spacing of 4.2 ms, and an echo train of length equal to 72. A 130-degree refocusing pulse was used to minimize the quantity of radio frequency (RF) power deposition. The acquisition time per image was just 430 ms per slice.

**Figure 1:**
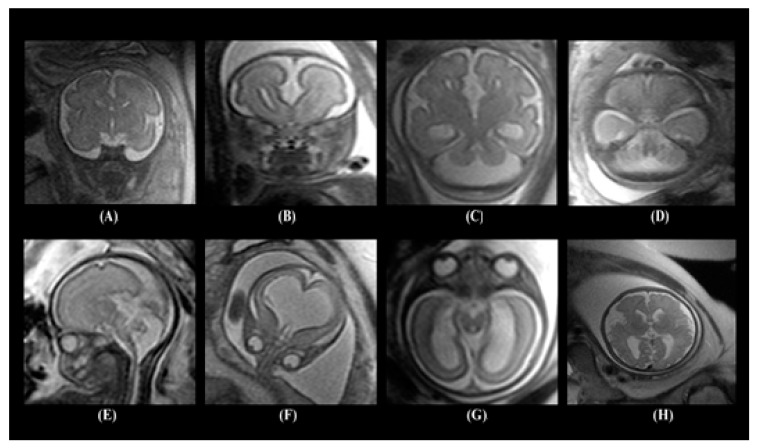
Images of different embryonic neurodevelopmental disorders: (**A**) normal embryonic brain, (**B**) agenesis of the corpus callosum, (**C**) colpocephaly, (**D**) mega-cisterna manga, (**E**) Dandy–Walker malformation, (**F**) agenesis of the septi pellucidi, (**G**) cerebellar hypoplasia, and (**H**) polymicrogyria.

The dataset contained several types of neurodevelopmental disorders such as cerebellar hypoplasia, Dandy–Walker variant/malformation, colpocephaly, agenesis of the corpus callosum, mega-cisterna magna, agenesis of the septi pellucidi, and polymicrogyria. Figure 1 shows samples of the MRI images including healthy embryonic brains and brains with neurodevelopmental disorders.

### 2.2. Deep Learning Techniques

The building blocks of artificial neural networks are known as “artificial neurons”. These artificial neurons simulate the behavior of human brain. Conventional neural networks contain fully connected (FC) layers consisting of a number of neurons. These neurons learn from a sequence of input data. They then propagate the learned information from the input to output layers through the hidden layer/layers by multiplying the weights between neurons of the FC layers. Afterward, the neurons of the output layer calculate the errors and use these errors to adjust weights of the previous layers [19]. Although this construction demonstrates a powerful design across various domains, it does not consider the privilege of the intrinsic spatial information contained in images. This network only studies configurations of activation as they are seen in a fixed order of the training data. As a solution to this limitation, other networks architectures were proposed known as deep learning (DL) [19,20,21]. DL architectures include recurrent neural networks (RNN) which are used for sequential data, restricted Boltzmann machines (RBM) and deep belief networks (DBN) which are commonly used in speech and image recognition, deep auto-encoders (DA) for unsupervised learning, and finally deep convolution neural networks (DCNN) employed for image/video segmentation and classification. The selection of network architectures and their parameters is made based on the given type of data and the type of the application or problem to be solved [21].

Among all DL architectures, the DCNN [22] is the commonly used structure for problems related to health informatics [23] and for specifically performing medical image classification [24]. Therefore, the DCNN structure was used in this article. DCNNs consist of a large number of deep layers; therefore, they are referred to as deep networks. The key units of DCNNs include convolutional layers, pooling layers, non-linear activation layers, FC layers, and the objective function loss layer. As an alternative to providing the entire image to each neuron, the DCNN convolves a filter of compact size with the input image. This leads to a group of neurons which only receive the regions of the input images corresponding to the size of the filter, followed by saving the position of the feature in the original image space. This can detect the location variations and returnable features by taking one full set of neurons in a hidden layer (called a feature map), which have similar parameters. The main advantage of this construction is the huge decrease in the number of parameters needed to be computed at each layer. Moreover, the feature map is now a spatial demonstration of the variables existing in the dataset [13,22,25]. DCNNs have several architectures; in this article, three of the state-of-the-art architectures are used, including AlexNet, GoogleNet, and ResNet 50.

#### 2.2.1. AlexNet DCNN Architecture

**Figure 2:**
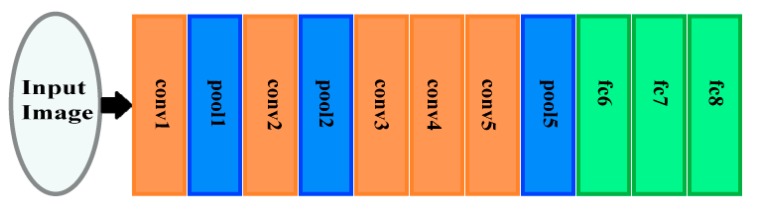
The archeictecture of AlexNet.

The AlexNet architecture is a DCNN which comprises five convolution layers, two fully connected (FC) layers, and three pooling layers. It has 60 million parameters [26]. Figure 2 displays the structure of the AlexNet architecture. The convolution layers of AlexNet are connected with a number of neurons with a dot product procedure between their weights and the area, which is linked to the input structure of an image [26]. Next, the pooling layers perform a down sampling operation.

The pooling layers are pool1, pool2, and pool5 as shown in Figure 2. These layers carry out a down sampling process throughout the spatial domain to decrease the amount of computational cost and to enhance the robustness [26,27]. These layers consist of pool1, pool2, and pool5 as displayed in Figure 1. They are succeeded by the fully connected layers FC6, FC7, and FC8, as presented in Figure 2. The neurons of these layers are fully connected to all neurons of the former layer, as done in conventional feed forward neural networks [26,28]. Table 1 shows the dimensions of different layers of the AlexNet DCNN.

#### 2.2.2. GoogleNet DCNN Architecture

**Figure 3:**
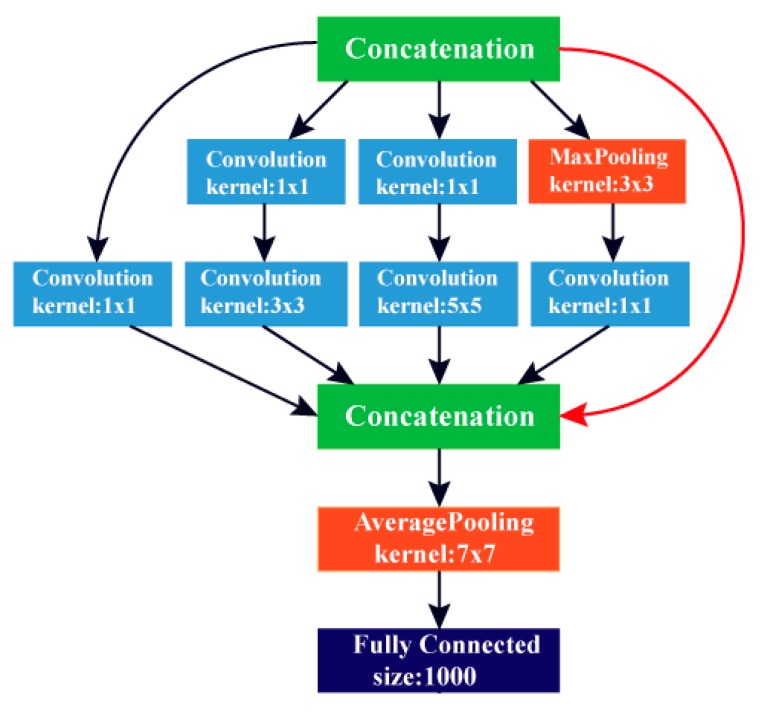
The architecture of the GoogleNet deep convolution neural network (DCNN).

GoogleNet is an architecture of DCNN. It was first presented by Szegedy et al. [29], featuring a construction with an effectively lower computational cost than AlexNet. The GoogleNet structure depends on the inception building block, which considers dropping the number of parameters in a CNN; thus, it is called Inception v1. Every layer of this network has nine inception elements and an FC layer. These inception elements are weighted upon each other, with a maximum pooling layer. The GoogleNet construction consists of 22 layers; therefore, it is considered extremely deeper than the ALexNet network. Despite the depth in its structure, it contains a lower number of parameters (almost 12 times lower) than AlexNet, which leads to a faster convergence. The GoogleNet architecture is shown in Figure 3. Table 2 shows the dimensions of different layers of the GoogleNet DCNN.

#### 2.2.3. ResNet 50 DCNN Architecture

**Figure 4:**
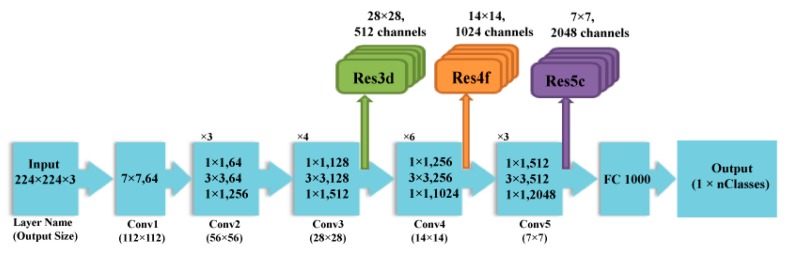
The architecture of the ResNet 50 DCNN.

ResNet is a more recent DCNN architecture that earned first place in the ILSVRC and COCO 2015 competition in terms of ImageNet Detection, ImageNet localization, Coco detection, and Coco segmentation [30]. It is a state-of-the-art DCNN that is commonly used network for many deep learning operations. It has efficient computational capabilities compared to other DCNNs such as AlextNet and Inception, which are more likely to fade and hardly converge with the increase in the number of layers [31]. The common method to resolve this issue is batch normalization; however, with the start of network convergence, the performance worsens rapidly and becomes flooded [32]. He et al. [30] offered a new solution for this problem, called the deep residual network (ResNet). This network relies on a deep residual learning structure which adds shortcuts (called residuals) within the layers of a conventional DCNN to pass over a few convolution layers at a time. These residuals enhance the performance of the network. They also speed up and facilitate the convergence process of the network even with a large number of deep convolution layers. Figure 4 shows the construction of the ResNet 50 architecture. Table 3 shows the dimensions of different layers of ResNet 50 DCNN.

### 2.3. Proposed Framework

**Figure 5:**
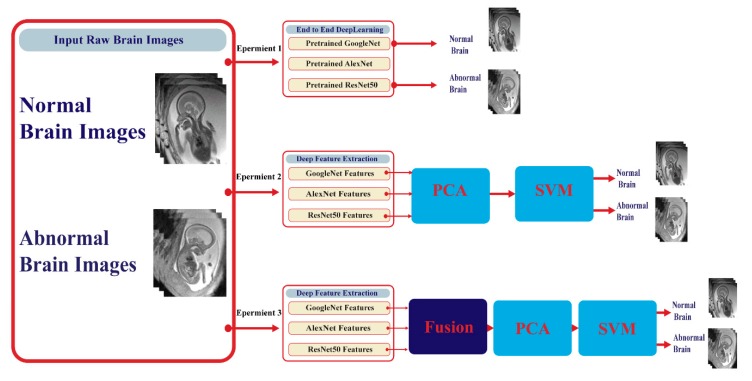
A block diagram of the proposed framework.

This article proposes a framework for automatic detection of embryonic neurodevelopmental disorders (ENDs) using deep learning techniques. The framework consists of four stages: transfer learning, deep feature extraction, feature reduction, and classification stages. Figure 5 shows a block diagram of the proposed framework. This framework corresponds to three experiments. In experiment I, each DCNN was used individually (end-to-end) to classify neurodevelopmental disorders. Deep features of each network were then separately extracted in experiment II, reduced using PCA, and fed to the support vector machine (SVM) for classification. In experiment III, every two to three deep features were combined together to form one feature vector for each image, again reduced by PCA and fed to SVM for classification. In the following subsections, the details of these stages are discussed in detail.

#### 2.3.1. Transfer Learning Stage

Training a DCNN is usually difficult due to overfitting and convergence problems, which need continuous modification of the network construction or parameters. This is done to make sure that all layers are learned with equivalent speed. The transfer learning technique provides a solution to these problems. Recently, it was widely adopted in analyzing images, specifically biomedical images. Transfer learning is the capability of achieving matches between different data or knowledge to ease the learning process of a novel task that has some joint characteristics. In other words, the pre-trained network has the ability to extract knowledge and information from huge data, and then use this information in other fields having the same classification problem. Definitely, in the biomedical area, finding datasets that are as huge and broadly labeled like the ImageNet dataset remains a challenge [20,24]. In the case of relatively small data or adequate data not being available, such as the dataset employed in this article (dataset contains 228 images), transfer learning is essential to overcome convergence and overfitting that would occur rapidly during the first few epochs. However, if it is pre-trained with a sufficient number of images, the learned representations from the data can be used to classify different classes of the same classification problem [31]. In transfer learning, DCNN models are pre-trained from a large image dataset like ImageNet or from a large dataset of a different medical domain. These pre-trained DCNNs are then used for a new medical task similar to the one at hand [24,33]. Therefore, in this stage, transfer learning was employed using several pre-trained DCNNs such as AlexNet, GoogleNet, and ResNet 50 architectures to detect END. These DCNN architectures were chosen as they are commonly used architectures for deep learning tasks. They are also the state-of-the-art DCNN models created in medical applications. As an alternative to constructing the DCNN from scratch, we began with formerly trained networks that were trained for similar classification problems. The DCNNs were pre-trained using the ImageNet dataset that consists of 1.2 million natural images classifying 1000 class labels. Afterward, the final fully connected (FC) layer was replaced by a novel layer suitable for classifying two class labels: normal and abnormal. Next, these models were used to detect ENDs and classify the brain as normal or abnormal.

#### 2.3.2. Deep Feature Extraction Stage

A pre-trained DCNN may be learnt from images; then, the outputs are taken from the FC layers of the network. For this reason, instead of using the FC layers of the DCNNs as classifiers, deep features were extracted from the first and second FC layers of the fine-tuned GoogleNet and AlexNet and from the average pooling layer of the fine-tuned ResNet 50. The number of deep features generated from each DCNN was 4096, 1024, and 2048 for AlexNet, GoogleNet, and ResNet 50, respectively.

#### 2.3.3. Feature Reduction Stage

Features extracted in the previous stage are of high dimensional space; therefore, a feature reduction process is needed to reduce the dimension of the feature space, reduce the complexity of the classification model, and lower the computational cost of the learning process. Principal component analysis (PCA) is a well-known feature reduction method that is widely used to shrink the data dimension by carrying out a covariance analysis between factors. PCA decreases the total observed features to a lower number of principal components. These principle components present the variance of the observed variables. PCA is usually applied to data when variables are highly correlated, and it is suitable for datasets in multiple dimensions [34].

#### 2.3.4. Classification Stage

In this stage, the classification process was carried out with three different scenarios. These scenarios were equivalent to the three experiments previously mentioned. The first scenario presents the use of the three DCNNs, namely AlexNet, GoogleNet, and ResNet, as classifiers (end-to-end deep learning process). Each pre-trained DCNN was constructed and trained separately and used as a classifier. As mentioned before, the pre-trained DCNN can be applied to images, and then the deep features are extracted from the FC layers of the network. These deep features can be used to train a distinct machine learning classifier which usually improves the performance of the classification task [33]. Therefore, in the second scenario, deep features extracted from each DCNN in the deep feature stage were used to separately train three SVM classifiers. Then, the three reduced feature sets produced from the PCA in the feature reduction stage were employed to individually construct three SVM classifiers. On the other hand, in the third scenario, different fused feature settings generated in the deep feature stage were used to construct several SVM classifiers to examine the best combination that influenced the classification accuracy. In the third scenario (experiment III), every two deep features were concatenated to form one feature vector for each image. This feature vector for each image was then concatenated to form a feature matrix, where the number of columns corresponded the dimension of the two deep features fused together, and the number of rows corresponded to the total number of images of the dataset. The same procedure was implemented when combining the three deep features together; however, the only difference was that the number of columns of the final feature matrix corresponded to the dimension of the three deep features fused together.

## 3. Experimental Set-Up

### 3.1. Data Augmentation

In order to construct an efficient detection model, a large dataset should be used. Training the model with a quite small dataset leads to overfitting during the learning process. This means that the model remembers the details of the training set and it does not generalize based on the validation or testing sets. Data augmentation is usually used to avoid the effect of overfitting [35]. It is a process which artificially creates new data from the existing training set using class-preserving perturbations of a dataset, which accordingly prevent overfitting which might happen when a fully connected layer inhabits most of the parameters. In this study, data augmentation was employed to generate new embryonic MRI images from the training data [12]. The augmentation techniques include flipping, translation, transformation, and rotating [31]. In this paper, each embryonic MRI image was translated in *x*- and *y*-directions with pixel range (−30, 30). Moreover, each original image was flipped to increase the size of the embryonic brain atlas dataset.

### 3.2. Parameter Setting

Some parameters were adjusted after fine-tuning the fully connected layer. The number of epochs and the initial learning rate for the three DCNNs were 20 and 10^−3^, respectively. The mini-batch size and validation frequency were 10 and three. The weight decay and momentum were set to 5 × 10^−4^ and 0.9, respectively. Other networks parameters were left with their default values. These configurations were to confirm that the parameters were fine-tuned for the detection of ENDs. The optimization algorithm used was the stochastic gradient descent with momentum (SGDM).

To assess the ability of the proposed framework to detect and classify ENDs, five-fold cross-validation was employed. This means that the embryonic brain atlas dataset was split into 80%/20% for training and validation. The SVM classifiers were trained with four folds and tested by the remaining fold. Therefore, the models were trained five times, and the testing accuracy was determined each time, then averaged. Note that the kernel function used for the SVM classifier was linear for experiments I and II; however, in experiment III, linear and quadratic kernels were used as they achieved better performance.

## 4. Evaluation Metrics

There are several evaluation tools to evaluate a classifier, including the accuracy, the sensitivity or true positive rate (TPR), and the specificity or true negative rate (TNR), as defined in Equations (1)–(3).

(1) $$Accuracy=\frac{TP+TN}{TP+TN+FP+FN}\;$$

(2) $$Sensitivity\left(TPR\right)=\frac{TP}{TP+FN}$$

(3) $$Specificity\left(TNR\right)=\frac{TN}{TN+FP}$$

## 5. Results

This paper proposes a framework for the automatic detection of embryonic neurodevelopmental disorders. The proposed approach consisted of three experiments. In experiment I, an end-to-end deep learning approach was implemented using three different structures of convolution neural networks (CNN), namely, GoogleNet, AlexNet, and ResNet 50. To reduce the complexity and computation time of the CNN models, deep features were extracted from each of the three CNNs in experiment II. These deep features were then used to train support vector machine classifiers to detect embryonic neurological disorders. Deep features extracted were of high dimensional space; therefore, principle component analysis (PCA) was employed to reduce their dimension. Experiment III combined these deep features extracted from the three CNNs in order to examine which combination of deep features influenced the accuracy of detection. The results of the proposed approach are illustrated in this section. To validate the efficiency of the proposed framework, the results were compared with related work done for ENDs that used the same dataset employed in the proposed work but used standard machine learning methods. This is because there was no related work that employed deep learning techniques for ENDs. The results were also evaluated against related work that investigated the use of deep learning techniques for the detection of neurodevelopmental disorders in an early age for premature infants and newborns.

### 5.1. Experiment I Results

This section presents the results of experiment I. In this experiment, three CNNs including GoogleNet, AlexNet, and ResNet architectures were constructed. Table 4 shows the results of these three networks. It is clear from the table that the GoogleNet accuracy was higher the other two network architectures.

### 5.2. Experiment II Results

**Figure 6:**
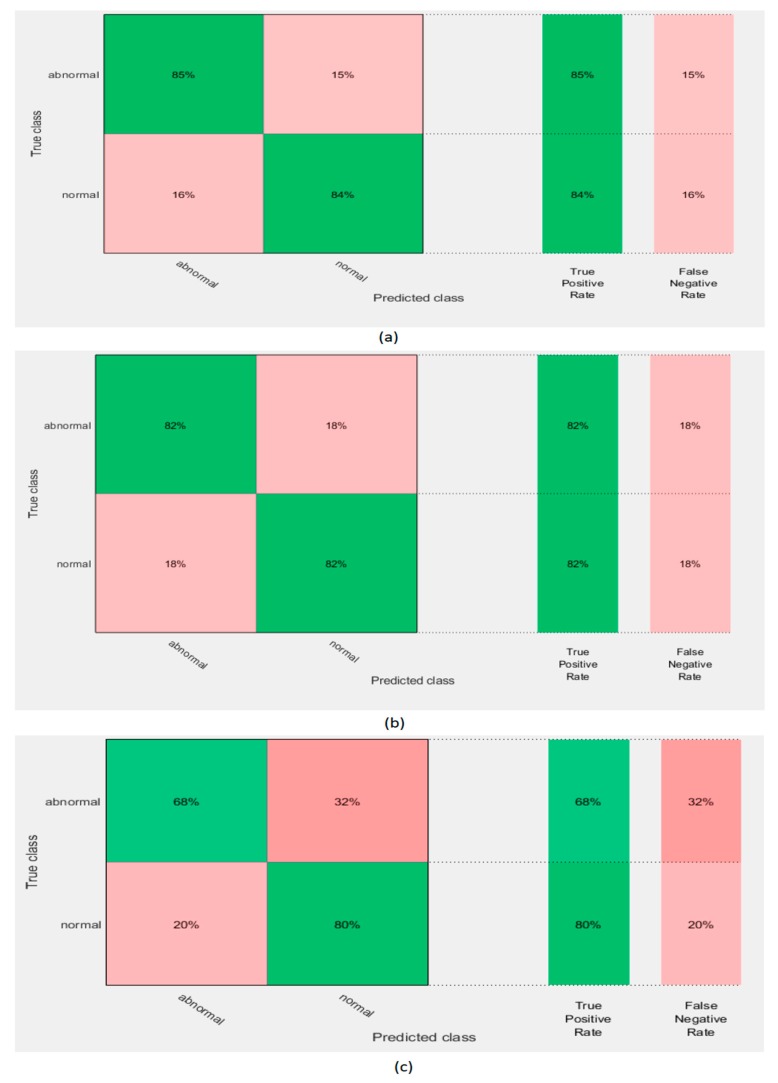
Confusion matrix of support vector machine (SVM) classifier trained with deep features extracted from the (**a**) GoogleNet DCNN,(**b**) AlexNet DCNN, and (**c**) ResNet 50 DCNN after principal component analysis (PCA).

This experiment extracted deep features from the three CNNs constructed in experiment I. It used these features to individually train three SVM classifiers. Table 5 shows the accuracy of the SVM classifiers trained with deep features extracted from the GoogleNet, AlexNet, and ResNet DCNNs before and after PCA. It is obvious that PCA increased the accuracy of the SVM classifiers trained with the three DCNNs. The highest accuracy of 83.8% was achieved using the GoogleNet DCNN. This accuracy was improved to 84.6% using PCA. Figure 6 shows the confusion matrix of the SVM classifier trained with the deep features of the GoogleNet, AlexNet, and ResNet 50 DCNNs after PCA. It shows that the highest sensitivity and specificity rates were 85% and 84% using GoogleNet.

**Figure 7:**
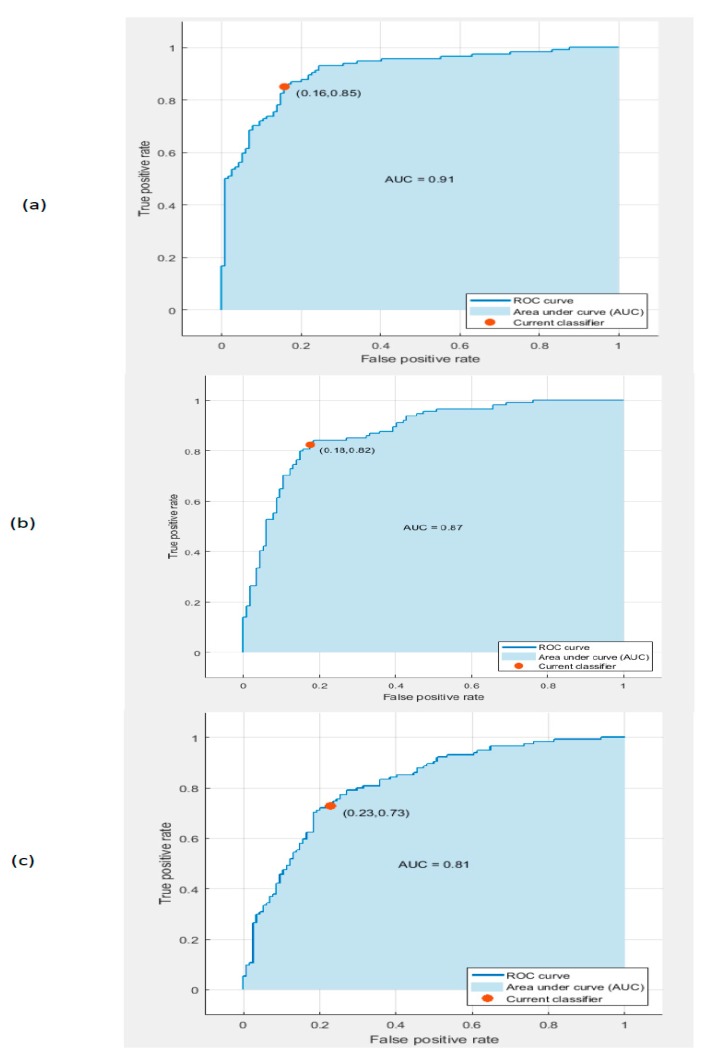
The receiver operating characteristic (ROC) curves for the linear SVM classifier trained with deep features extracted from the (**a**) GoogleNet DCNN, (**b**) AlexNet DCNN, and (**c**) ResNet 50 DCNN after PCA.

The receiver operating characteristic (ROC) curves for the SVM classifier trained with deep features extracted from the Google Net, AlexNet, and ResNet 50 DCNNs after PCA are shown in Figure 7. The figure shows that the areas under the curve (AUCs) for the GoogleNet, AlexNet, and ResNet 50 DCNNs were 0.91, 0.87, and 0.81, respectively.

### 5.3. Experiment III Results

**Figure 8:**
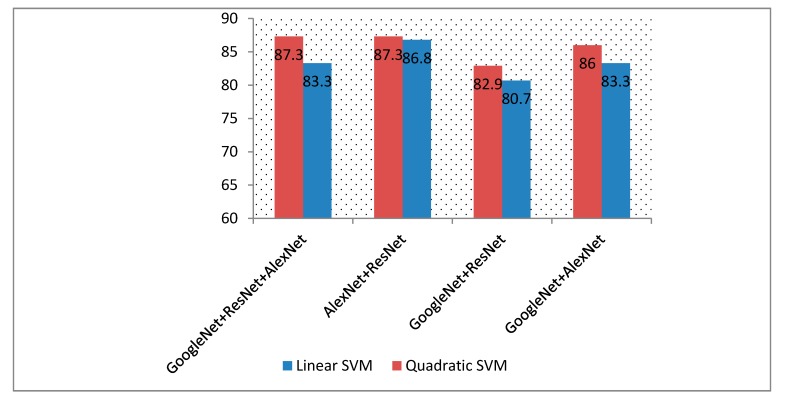
A comparision between the accuracies of different combinations of deep features extracted from different DCNNs.

This experiment illustrated the results of combining different deep features extracted from the GoogleNet, AlexNet, and ResNet DCNNs. Figure 8 shows the accuracy of various combinations of these deep features used to train SVM classifiers.

Table 6 represents the classification accuracy of the linear SVM with and without PCA. As shown in Figure 8, fusing the deep features extracted from the DCNNs improved the classification accuracy of the SVM classifiers. The highest accuracy achieved was 88.6% using quadratic SVM classifiers trained with deep features extracted from AlexNet and ResNet 50 together. An accuracy of 87.7% was also achieved by the quadratic SVM trained with GoogleNet, AlexNet, and ResNet 50 deep features. In both cases, the performance of the SVM was higher than when using individual deep features to train the SVM, as shown in Table 2 (the accuracies achieved were 84.2%, 82%, and 75% for GoogleNet, AlexNet, and ResNet, respectively).

**Figure 9:**
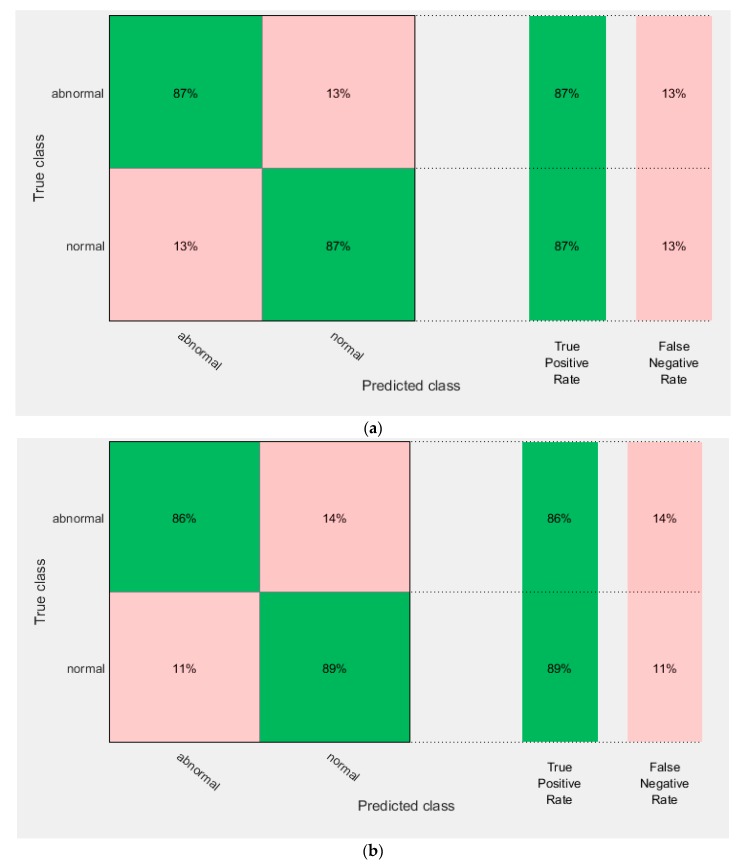
The confusion matrix of AlexNet + ResNet 50 fused features using (**a**) the linear SVM, and (**b**) the quadratic SVM.

**Figure 10:**
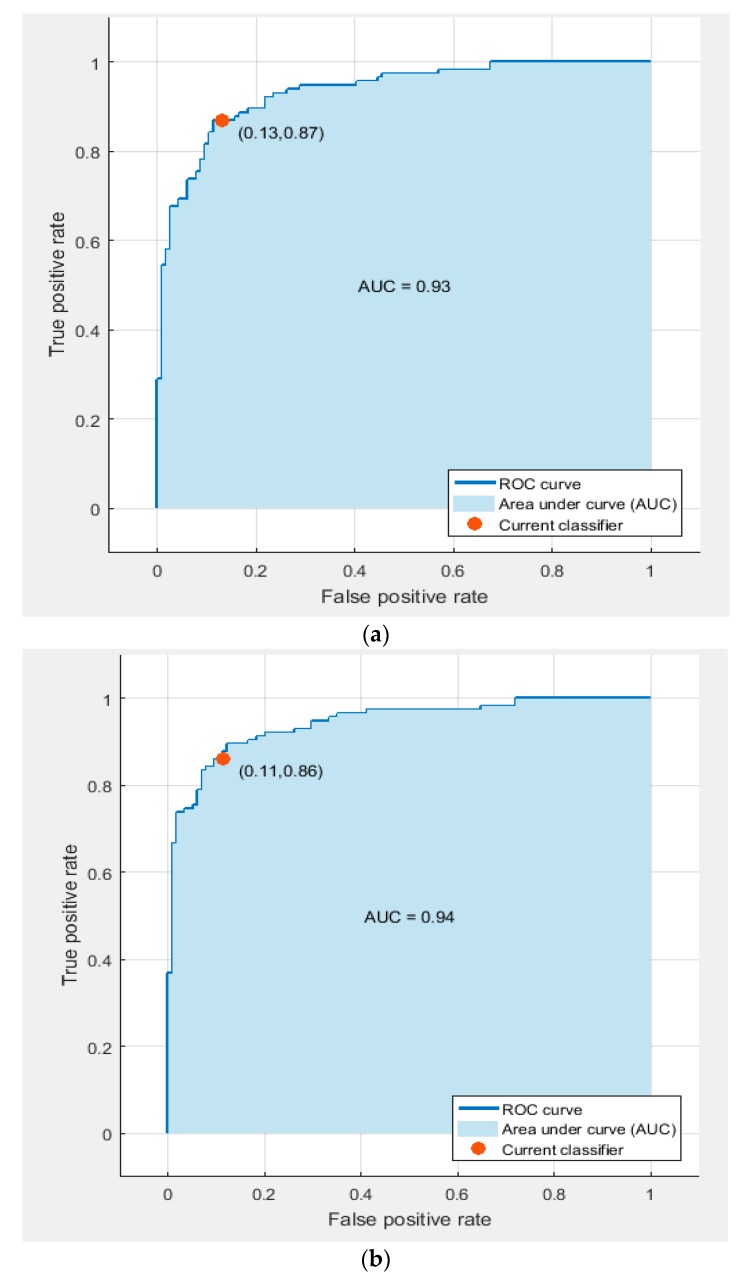
ROC curves and areas under the curve (AUCs) for AlexNet and ResNet 50 fused faetures used to train (**a**) the linear SVM classifier, and (**b**) the quadratic SVM classifier.

Figure 9 represents the confusion matrix for the AlexNet and ResNet 50 fused features, which achieved the highest classification accuracy using linear and quadratic SVM classifiers. Figure 10 shows the ROC curves and AUCs for these feature combinations used to train the linear and quadratic SVM.

### 5.4. Comparison with Related Work

This section presents a comparative study between the results of the proposed framework and other recent related work for END detection using the same dataset employed in this study. Table 7 represents this comparison. As can be seen from the table, recent related work used conventional machine learning methods; however, as stated before, they had several limitations. Deep learning methods can overcome these limitations and construct a powerful classification model without the need for image enhancement, segmentation, and feature extraction. Therefore, it was used in this paper. We used the raw data to construct a classification model capable of detecting ENDs. The results in the table show that the proposed framework is competitive with other methods based on standard machine learning techniques. The highest accuracy achieved using the proposed framework was 88.6% which was greater than that achieved in Reference [10], but slightly lower than that achieved in Reference [11].

## 6. Discussion

Early detection of NDs in embryos is vital; however, it is a complicated process due to several reasons. The complication arises during the MRI scanning process of embryos. This is due to the small embryonic brain size and the continuous motion of the embryo inside the utero. Also, parts of the parental body appear in the MRI images in addition to the embryonic body. Moreover, tissue contrast variations are observed in utero for older GA [6]. Recently, machine learning and specifically deep learning methods attracted a lot of researchers working in the medical field to help solve medical problems. The use of such techniques can assist neurologists and facilitate the detection and classification of ENDs. However, the use of machine learning and deep learning techniques was not extensively explored. There are a limited number of research articles [8,9,10,11] that investigated the use of such techniques for detecting ENDs. These articles utilized conventional machine learning methods for feature extraction and the classification of ENDs. The feature extraction techniques employed in these articles were handcrafted and depended on the delineation of brain structures, which had several limitations and drawbacks [12,13]. Deep learning approaches are another class of machine learning methods which are more favorable as they are capable of inferring an optimum explication from the raw images without image enhancement, segmentation, and feature extraction processes, leading to a better classification process and lower complexity [15].

In this paper, a new framework based on deep learning methods was proposed for detecting ENDs. To the best of our knowledge, this is the first study that examined the use of deep learning approaches for detecting ENDs. The new proposed framework consists of four stages: transfer learning, deep feature extraction, feature reduction, and classification stages. In the transfer learning stage, different CNN architectures, such as AlexNet, GoogleNet, and ResNet 50, were constructed for detecting ENDs. Deep features were then extracted from each DCNN in the deep feature extraction stage. These features were of large dimension; therefore, a feature reduction process was carried out using PCA to lower their dimension in the feature reduction stage. In the classification stage, several SVM classifiers were built to detect ENDs using deep features separately extracted from each DCNN. Afterward, several deep feature combinations were used to train different SVM classifiers to test the effect of feature fusion on the classification accuracy and to select the best combination of deep features.

This study conducted three experiments. Experiment I executed an end-to-end deep learning method via three different structures of CNNs to detect ENDs. in experiment II, deep features were extracted from each DCNN structure and used individually to train different SVM classifiers. Afterward, PCA was employed to separately reduce the deep feature dimensions and then construct several SVM classifiers. In experiment III, deep feature fusion was performed to determine its impact on the classification performance and to select the best combination of deep features which enhanced the performance. As can be seen in Table 1, the highest accuracy of 77.9% was achieved using the GoogleNet DCNN. This accuracy was improved in experiment II, as shown in Table 2, to 83.8% using the SVM classifier trained with deep features extracted from the GoogleNet DCNN. It was further increased to 84.6% when the PCA feature reduction method was applied. This indicated that exchanging the FC layers of the DCNN with SVM classifiers improved the classification accuracy. An addition improvement in the classification accuracy was noted in experiment III when using deep feature fusion. The highest accuracy achieved was 88.6% using a combination of deep features extracted from AlexNet and ResNet 50 DCNNs. An accuracy of 87.7%was also achieved using deep features of GoogleNet, AlexNet, and ResNet 50 DCNNs. This shows that the feature fusion can impact classification accuracy and improve the performance of SVM classifiers.

The advantage of the proposed framework is using deep learning techniques on raw data without the need for image enhancement, segmentation, and feature extraction, thereby achieving good classification accuracy compared to other related work that used conventional machine learning methods for ENDs. Moreover, it was capable of detecting ENDs with images containing embryos of various GAs, not only one age.

Deep learning (DL) techniques are the newest class of machine learning techniques. Recently, DL methods were used extensively, as they can overcome limitations of classical machine learning methods. DL methods showed their superiority over classical machine learning methods in most cases. As long as the amount of data increases, DCNNs outperform standard machine learning techniques. Our approach outperformed the method proposed in Reference [10]. However, in some cases, when the dataset was relatively small, standard machine learning techniques might outperform deep learning methods [36,37], which was the case when comparing with Reference [11]. Reference [11] applied handcrafted feature extraction methods such as a Gabor filter and Gray Level Co-occurrence Matrix (GLCM). Although these feature extraction methods produced higher results, they are time-consuming, they need high computational cost, and they are arduous and prone to inter- and intra-rater inconsistency, or compound pre-processing of MRI images [12,13]. DL methods reduce such limitations.

Certainly, in the biomedical area, finding huge datasets that are as completely labeled as the ImageNet dataset remains a challenge [20,24]. When adequate data are not available, as for the dataset employed in our article, transfer learning is essential in order to overcome overfitting and convergence problems that would occur due to a small dataset size. In transfer learning, DCNN models are pre-trained from a large image dataset like ImageNet or from a large dataset from a different medical domain. These pre-trained DCNNs are then used for a new medical task similar to the one at hand [24]. Therefore, we employed pre-trained networks instead of constructing our own CNN, as the dataset used in the proposed framework was relatively small, which increased chances of overfitting and convergence problems.

Table 8 shows the difference between elapsed times of the AlexNet, Google Net, and ResNet 50 DCNNs, which were 5 min 57 s, 10 min 29 s, and 6 min 57 s. As can be noted, although ResNet is a dense network, it had effective computational abilities compared to AlextNet (less dense) and GoogleNet, which were more likely to fade and hardly converge with the increase in the number of layers [31]. The experiments were performed on am Intel^®^ CORE™ I7 processor and NVIDIA GETFORCE GTX 1050, Windows 10, 64 bit with 16 GB of random-access memory (RAM). The software used to implement the experiments was Matlab R2018b.

**Figure 11:**
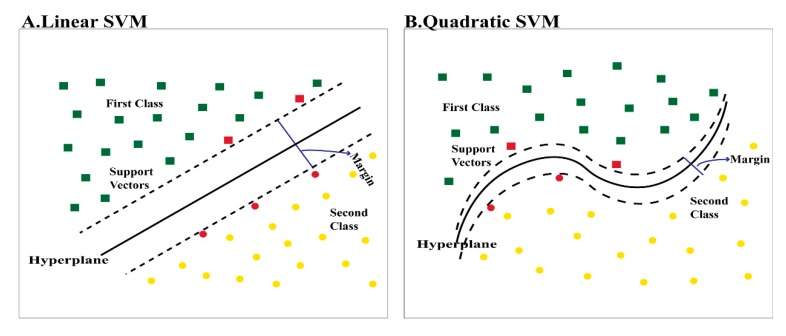
The difference between (**A**) a linear kernal and (**B**) a quadratic kernel.

SVM classifiers were used to classify ENDs in this article. The concept of the SVM classifier is to change an input vector which is not linearly separable using a hyperplane into a higher-dimensional feature space that is able to linearly distinguish between classes of input data to ease the classification process. The procedure is achieved using a kernel function which maps the similarity between the input vector and the new higher-dimension feature space. A linear kernel is usually used when that dataset is simply separated by a linear line. However, a quadratic kernel is a nonlinear kernel used when the dataset is complex and not linearly separable. Quadratic kernels may increase the accuracy. Other advantages of quadratic kernels include having elegant mathematical tractability and direct geometric interpretation [38]. The dataset used was not linearly separable; therefore, the quadratic kernel produced better results than that of the linear kernel. Kernel equations used to transform the feature space into a higher dimensional feature space are shown below. Figure 11 displays the difference between linear and quadratic kernels.

(4) $$K_{linear}=y*x+b$$

(5) $$K_{Quadratic}={\left(y*x+b\right)}^{2}$$

where *x* and *y* are the input *n*-dimensional feature values, *b* is the kernel parameter, and *K* is the Kernel function.

PCA is commonly used to reduce the high-dimensional space of the deep features extracted using DCNNs. It was used extensively in References [39,40,41,42,43,44,45,46] to lower the dimension of deep features used in training SVM classifiers and to also lower the SVM’s complexity. SVM classifiers have very effective performance in classification tasks with limited training samples. As can be seen in experiments II and III, the accuracy increased after using PCA in most cases.

As mentioned before, the literature showed that the work done for ENDs is limited to a few research articles. These articles used standard machine learning techniques for detecting ENDs. To the best of our knowledge, the use of deep learning techniques for ENDs was not studied. We did not find any article that used deep learning for ENDs to allow a comparison with our work. Therefore, we only compared recent related work based on conventional machine learning methods for END detection.

## 7. Conclusions

Neurodevelopmental disorders are dangerous defects affecting the human brain and influencing the natural development of the central nervous system. These defects lead to malfunction of the brain activity, which corresponds to obvious neuropsychiatric deficiencies, learning complications, language or non-verbal communication difficulties, or motor function disability [2]. Recent studies showed that three out of 1000 pregnant women have embryos with NDs. Early discovery of embryonic NDs is essential. Recently, machine learning and specifically deep learning methods were used extensively to solve medical problems like ND detection. Primary detection using such techniques has several benefits. Firstly, they can help doctors precisely identify the brain deficiency before the infant is born, allowing them to select the appropriate follow-up and treatment plans. Moreover, families will be ready and aware in terms of managing the defect. Correspondingly, the quality of diagnosis and health management will be enhanced. It will also reduce the chances of progression of the neurological disorder after the birth of the embryo.

Even though the early discovery of ENDs is a very important research topic, little work was done to explore this area. Such work was limited to studies that used conventional machine learning techniques; to the best of our knowledge, this is the first study which investigated the use of deep learning methods. This paper proposed a framework based on deep learning techniques for the early detection of ENDs. It consisted of four stages: transfer learning, deep feature extraction, feature reduction, and classification stages. The study conducted three experiments. In the first experiment, an end-to-end deep learning method was implemented via three different constructions of CNN. In experiment II, deep features were extracted from the FC layer of each DCNN. They were then employed to separately train different SVM classifiers. After that, these features were reduced using PCA and then used to construct several SVM classifiers. On the other hand, deep features were combined to examine their impact on the classification performance and to select the best combination of deep features which enhanced the performance.

The results of the proposed framework indicate that it is capable of detecting ENDs with good classification accuracy. The accuracy of the proposed framework is competitive with recent related work for END detection. Therefore, it can be used by neurologists to facilitate the diagnosis process of embryonic brain deficiencies. It will also ease the treatment and follow-up management plans and enable parents to understand the nature of the defect. This will consequently decrease the chances of development of the NDs after the birth of the embryo and enhance the quality of health management.

Future work will focus on using other deep learning architectures and constructing a new DCNN. Also, fusing more deep features and combining such features with other handcrafted features will be investigated. Moreover, collecting more images for embryos will be done. Furthermore, feature selection methods will be studied to try to improve the classification performance of the classification models.

## Figures and Tables

**Table 1:** The dimensions of different layers of the AlexNet deep convolution neural network (DCNN).

Layer Label	Specifications		Output Dimension
Input layer			227 × 227 × 3
Convolution layer 1	Filter size	11 × 11	55 × 55 × 96
	Stride	4	
	Padding	0	
Pooling layer 1	Pooling size	3 × 3	27 × 27 × 96
	Stride	2	
Convolution layer 2	Filter size	5 × 5	27 × 27 × 256
	Stride	1	
Pooling layer 2	Pooling size	3 × 3	13 × 13 × 256
	Stride	2	
Convolution layer 3	Filter size	3 × 3	13 × 13 × 384
	Stride	1	
Convolution 4	Filter Size	3 × 3	13 × 13 × 384
	Stride	1	
Convolution layer5	Filter size	3 × 3	13 × 13 × 256
	Stride	1	
Pooling layer 5	Pooling size	3 × 3	6 × 6 × 256
	Stride	2	
FC6 layer			4096 × 2
FC7 layer			4096 × 2
FC8 layer			1000 × 2

FC—fully connected.

**Table 2:** Different layers dimension of a GoogleNet DCNN.

Layer Label	Filter Dimension	Stride	Output Dimension
Input layers			224 × 224 × 3
Convolution layer 1	7 × 7	2	112 × 112 × 64
Pooling layer 1	3 × 3	2	56 × 56 × 64
Convolution layer 2	3 × 3	1	56 × 56 × 192
Pooling layer 2	3 × 3	2	28 × 28 × 192
Inception layer (3a)	-	-	28 × 28 × 256
Inception layer (3b)	-	-	28 × 28 × 480
Pooling layer 3	3 × 3	2	14 × 14 × 480
Inception layer (4a)	-	-	14 × 14 × 512
Inception layer (4b)	-	-	14 × 14 × 512
Inception layer (4c)	-	-	14 × 14 × 512
Inception layer (4d)	-	-	14 × 14 × 528
Inception layer (4e)	-	-	14 × 14 × 832
Pooling layer 4	3 × 3	2	7 × 7 × 832
Inception layer (5a)	-	-	7 × 7 × 832
Inception layer (5b)	-	-	7 × 7 × 1024
Average pooling layer	7 × 7	1	1 × 1 × 1024
FC layer			1024 × 2

**Table 3:** Different layers dimensions of the ResNet 50 DCNN.

Layer Label	Input Layer Dimension	Output Dimension
Input Layer		227 × 227 × 3
Conv1	112 × 112 × 64	Filter size = 7 × 7 Number of filters = 64 Stride = 2 Padding = 3
pool1	56 × 56 × 64	Pooling size = 3 × 3 Stride = 2
Conv2_x	56 × 56 × 64	$\left[\begin{array}{cc} 1\;\times 1. & 64\\ 3\;\times 3. & 64\\ 1\;\times 1. & 256 \end{array}\right]$ ×3
Conv3_x	28 × 28 × 128	$\left[\begin{array}{cc} 1\;\times 1. & 128\\ 3\;\times 3. & 128\\ 1\;\times 1. & 512 \end{array}\right]$ ×4
Conv4_x	14 × 14 × 256	$\left[\begin{array}{cc} 1\;\times 1. & 256\\ 3\;\times 3. & 256\\ 1\;\times 1. & 1024 \end{array}\right]$ ×6
Conv5_x	7 × 7 × 512	$\left[\begin{array}{cc} 1\;\times 1. & 512\\ 3\;\times 3. & 512\\ 1\;\times 1. & 2048 \end{array}\right]$ ×3
Average pooling		Pool size = 7 × 7 Stride = 7
		1 × 1 × 2048
FC layer		2 (2048 × 2)

**Table 4:** Performance metrics of GoogleNet, AlexNet, and ResNet DCNNs.

DCNN	Accuracy (%)	Sensitivity (%)	Specificity (%)
GoogleNet	77.9	79.4	76.5
AlexNet	73.5	85.3	61.8
ResNet 50	76.5	82.4	70.6

**Table 5:** The accuracy of the support vector machine (SVM) classifiers trained with GoogleNet, AlexNet, and ResNet 50 DCNNs. PCA—principal component analysis.

SVM Trained with Deep Features	Accuracy (%) of Deep Features without PCA	Accuracy (%) of Deep Features with PCA
	Linear SVM	
GoogleNet	83.8	84.6
AlexNet	81.1	82.0
ResNet 50	75.0	75.0
	Quadratic SVM	
GoogleNet	79.4	78.9
AlexNet	84.6	85.5
ResNet 50	79.8	79.8

**Table 6:** A comparison between the accuracies of the linear SVM classifier trained with several deep features extracted from different DCNNs.

DCNN	Accuracy (%) of Deep Features without PCA	Accuracy (%) of Deep Features with PCA
	Linear SVM	
Google + ResNet 50	80.7	86
Google + AlexNet	83.3	82.0
AlexNet + ResNet 50	86.3	87.2
The three DCNNs	83.3	84.2
	Quadratic SVM	
Google + ResNet 50	82.9	83.8
Google + AlexNet	86	86.8
AlexNet + ResNet 50	87.3	88.6
The three DCNNs	87.3	87.7

**Table 7:** A comparision between the accuracy of the proposed framework and recent related work.

Article	Feature Extraction	Classifier	Accuracy (ACC)
[10]	Discrete wavelet transform + statistical features	Linear Discriminant Analysis (LDA)	79%
		SVM	79%
		K Nearest Neighbor (KNN)	73%
		Ensemble Subspace Discriminates	80%
[11]	Gabor filter + Gray Level Co-occurrence Matrix (GLCM) + PCA	Diagonal Quadratic Discriminant Analysis (DQDA)	92%
		Neural networks	93%
		Naïve Bayes	91.63%
		Random forest	90.3%
The proposed framework	Deep features:	Linear SVM	84.2%
	GoogleNet + AlexNet + ResNet 50	Quadratic SVM	87.7%
	Deep features:	Linear SVM	87.2%
	AlexNet + ResNet 50	Quadratic SVM	88.6%
	Deep features:	Linear SVM	86%
	GoogleNet + ResNet 50	Quadratic SVM	83.8%
	Deep features:	Linear SVM	82%
	GoogleNet + AlexNet	Quadratic SVM	86.8%

**Table 8:** The elapsed time of AlexNet, GoogleNet, and ResNet 50 DCNNs.

DCNN	Training Time
AlexNet	5 min 57 s
GoogleNet	10mins 29 s
ResNet 50	6 min 25 s
